# Implanted haemodynamic telemonitoring devices to guide management of heart failure: a review and meta-analysis of randomised trials

**DOI:** 10.1007/s00392-022-02104-0

**Published:** 2022-10-14

**Authors:** Antonio Iaconelli, Pierpaolo Pellicori, Elisabetta Caiazzo, Asma O. M. Rezig, Dario Bruzzese, Pasquale Maffia, John G. F. Cleland

**Affiliations:** 1 School of Cardiovascular and Metabolic Health, University of Glasgow, Glasgow, UK; 2 Department of Cardiovascular Medicine, Fondazione Policlinico Universitario A. Gemelli IRCCS, 00168 Rome, Italy; 3 School of Infection and Immunity, College of Medical, Veterinary and Life Sciences, University of Glasgow, Glasgow, UK; 4 Department of Pharmacy, School of Medicine and Surgery, University of Naples Federico II, Naples, Italy; 5 Department of Public Health, School of Medicine and Surgery, University of Naples Federico II, Naples, Italy

**Keywords:** Tele-monitoring, Pulmonary hypertension, Implantable devices, Heart failure

## Abstract

**Background and aims:**

Congestion is a key driver of morbidity and mortality in heart failure. Implanted haemodynamic monitoring devices might allow early identification and management of congestion. Here, we provide a state-of-the-art review of implanted haemodynamic monitoring devices for patients with heart failure, including a meta-analysis of randomised trials.

**Methods and results:**

We did a systematic search for pre-print and published trials in Medline, Embase, and the Cochrane Central Register of Controlled Trials (CENTRAL) on the 22nd of September 2021. We included randomised trials that compared management with or without information from implanted haemodynamic monitoring devices for patients with heart failure. Outcomes selected were hospitalisation for heart failure and all-cause mortality. Changes in treatment associated with haemodynamic monitoring resulted in only a small reduction in mean pulmonary artery pressure (typically < 1 mmHg as a daily average), which generally remained much greater than 20 mmHg. Haemodynamic monitoring reduced hospitalisations for heart failure (HR 0.75; 95% CI 0.58–0.96; *p* = 0.03) but not mortality (RR 0.92; 95% CI 0.68–1.26; *p* = 0.48).

**Conclusions:**

Haemodynamic monitoring for patients with heart failure may reduce the risk of hospitalization for heart failure but this has not yet translated into a reduction in mortality, perhaps because the duration of trials was too short or the reduction in pulmonary artery pressure was not sufficiently large. The efficacy and safety of aiming for larger reductions in pulmonary artery pressure should be explored.

**Graphical abstract:**

**Figure:**
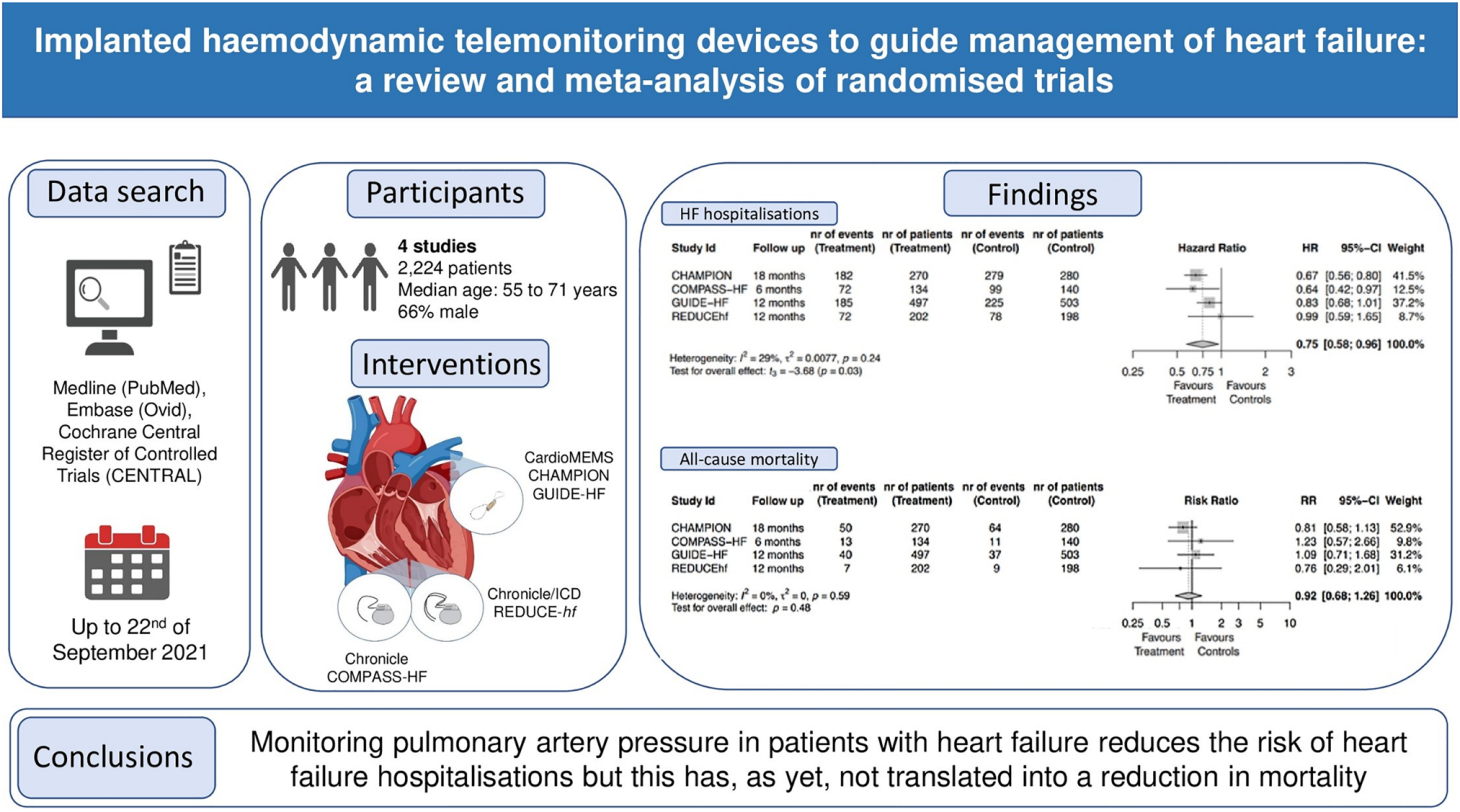
After selecting key words, a systematic review for implanted haemodynamic telemonitoring devices was performed in different dataset and 4 randomised clinical trials were identified and included in this meta-analysis. Three different devices (Chronicle, Chronicle/ICD and CardioMEMS) were tested. All-cause mortality and total heart failure hospitalisations were selected as outcomes. No reduction in all-cause mortality rate was reported but a potential benefit on total heart failure hospitalisation was identified.

**Supplementary Information:**

The online version contains supplementary material available at 10.1007/s00392-022-02104-0.

## Introduction

Heart failure can be defined as cardiac dysfunction associated with interstitial or intravascular water and salt retention, otherwise known as congestion. Congestion is not only a cause of symptoms and signs but may also cause cardiac dysfunction, remodelling and arrhythmias, which are all associated with a poorer prognosis [1]_._ Identifying, quantifying and treating congestion at an early stage is a key task for good management of heart failure, but it is currently done sub-optimally.

Symptoms and signs have traditionally been used to guide therapy but are not specific for heart failure and may only be obvious once decompensation is severe [2]. High plasma concentrations of natriuretic peptides correlate with symptoms and with the severity of cardiac dysfunction [1] and are associated with an increased risk of hospitalisation and death due to heart failure; although measurement of natriuretic peptides facilitates initial diagnosis, their serial evaluation has not been shown—convincingly—to improve heart failure management [3]. The requirement for more than a drop of blood for point-of-care measurement makes monitoring by patients at home difficult [4].

Echocardiography is not only widely available and frequently used for non-invasive, real-time assessment of cardiac structure and function but can also provide information on congestion. However, it requires expertise to acquire and interpret images, and there is little evidence, as yet, that serial ultrasound can be used to guide diuretic treatment. There is also a lack of robust evidence that other non-invasive approaches to estimate the amount of body water and its distribution, such as weighing scales, bio-impedance or remote dielectric sensing, improve management and outcomes for patients with heart failure [5].

Elevated pulmonary artery (PA) or right ventricular pressures also reflect congestion and identify patients with heart failure who are at greater risk of hospitalization or death [6]. Recently, implantable miniaturised sensors have been developed and tested to assist clinicians in the management of patients with symptomatic heart failure, allowing treatments to be haemodynamically tailored for each patient individually in the hope that this will improve well-being, reduce hospitalisation and, hopefully, increase longevity [7].

In this manuscript, we summarise the rationale and the current state-of-the-art of implanted haemodynamic monitoring devices for patients with heart failure. We also conducted a systematic review and meta-analysis of randomised trials to investigate the effects of this strategy on heart failure hospitalisations and mortality in this population.

## Pulmonary hypertension and heart failure: a vicious circle

Recently, updated guidelines reduced the threshold for diagnosing PH from a mean PAP of 25 mmHg down to 20 mmHg, assessed at rest during a right heart catheterisation (RHC) [8]. However, the median value for a resting PAPm in a healthy population is approximately 15 mmHg, with a normal range of about 11–17 mmHg [9]. Data from a broad range of patients, mostly men, in the VA-CART programme (> 20,000 patients, 97% men, 2473 with heart failure) found that the risk of hospitalisation and mortality starts to increase, progressively, when an invasively measured PAPm exceeds 19 mmHg [10].

In routine clinical practice, ultrasound is often used to identify PH, combining information from tricuspid regurgitation (TR) peak velocity and inferior vena cava diameter and collapsibility to estimate PA systolic pressure (PAPs) [11]; an echocardiogram will also help identify causes of PH, such as mitral valve or left ventricular disease. PAPs measured by ultrasound generally correlate well with invasively measured values [12]. However, peak TR velocity may underestimate PAPs when tricuspid regurgitation is severe or in case of right ventricular dysfunction, necessitating invasive assessment to confirm a diagnosis or quantify the severity of PH [8].

Persistently elevated left atrial pressures, due to left ventricular dysfunction or mitral valve disease, are transmitted backwards to the pulmonary circulation, leading to a rise of pulmonary artery pressure (PAP) [13]. At first, PA pressures may be largely dictated by left atrial pressure, rising only with exercise or episodes of decompensation. However, over time, increases in pulmonary vascular tone and hypertrophy of the pre-capillary pulmonary vascular smooth muscle may develop. Pathophysiologically, this may protect the pulmonary capillaries from increases in pulmonary arterial, although not pulmonary venous, pressure. Eventually, vascular remodelling may lead to relatively fixed pulmonary hypertension that is independent of left atrial pressure and may be relatively unresponsive to pulmonary vasodilators, which may even have deleterious effects by increasing perfusion to poorly aerated lung regions (ventilation perfusion mismatch) [14–16]. An elevated PAP increases the load on the right ventricle (RV), which may cause dilation and dysfunction, leading to tricuspid regurgitation and increasing systemic venous congestion. Once this happens, the risk of decompensation, hospital admission and death increases substantially [17, 18].

PH is common in patients with heart failure, but its reported prevalence depends on the criteria used to define it and the severity of heart failure [19–21]. Perhaps all patients with chronic heart failure have some PH and it is not really a question of prevalence but only of severity. For most patients with heart failure, the severity of PH at rest is mild. A study in a broad population of heart failure, defining PH as a PAPs of > 45 mmHg by ultrasound, suggested a prevalence of PH less than 10% [6, 22]. Defining PH as a PAPs ≥ 35 mmHg provides much higher estimates of prevalence ranging from about 30–50% amongst patients with heart failure and preserved left ventricular ejection fraction (HFpEF) [6, 17, 23, 24]. Similarly, a series of reports suggests that most patients with heart failure and reduced ejection fraction (HFrEF) have PH, with the prevalence varying by the stringency of the diagnostic criteria for PH [20, 25–28] (Table 1).

**Table 1:**
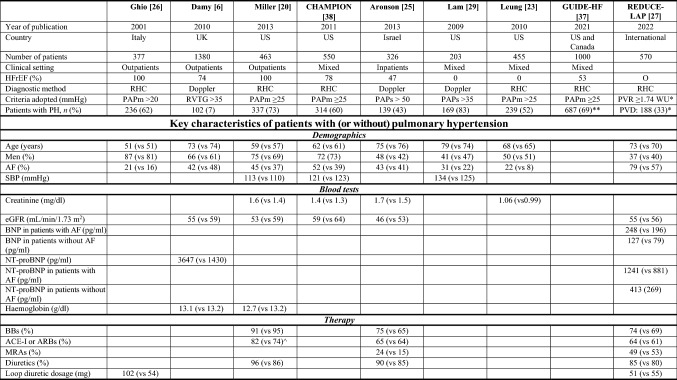
Prevalence of pulmonary hypertension in cohorts or trials of patients with heart failure

*ACE-I/ARBs* angiotensin-converting enzyme inhibitors and angiotensin receptor blockers, *AF* atrial fibrillation, *BBs* beta-blockers, *BNP* B-type natriuretic peptides, *eGFR* estimated glomerular filtration rate, *HFrEF* heart failure with reduced ejection fraction, *MRAs* mineralocorticoid antagonists, *NT*-*proBNP* amino-terminal pro-brain natriuretic peptide, *PAPm* pulmonary artery mean pressure, *PAPs* pulmonary artery systolic pressures, *PH* pulmonary hypertension, *PVD* pulmonary vascular disease, *PVR* pulmonary vascular resistance, *RHC* right heart catheterization, *RVTG* right ventricular tricuspid gradient, *SBP* systolic blood pressure, *UK* United Kingdom, *US* United States

^a^Patients with pulmonary vascular disease are defined as PVR ≥ 1.74 WU at 20 W of exercise, measured prior to the randomisation

^b^The prevalence rate of PH in the GUIDE-HF is not provided by the authors: the value reported in this table is an estimate obtained considering the average of PAPm, the standard deviation and the number of specimens of the study population, assuming a normal distribution

^c^This data refers only to ACE-I. Data are reported as *n* (%) and mean. Data on the REDUCE-LAP section are reported as *n* (%) and median

Patients with PH generally have more severe heart failure, are older [23, 29] and are more likely to have atrial fibrillation [20, 23, 26, 28], poorer renal function [20, 25], higher plasma concentrations of natriuretic peptides [20, 25] and to be treated with loop diuretics [6, 20, 22, 25] (Table 1). Indeed, patients who do not have distinctly elevated plasma concentrations of natriuretic peptides often do not have sufficient tricuspid regurgitation to measure velocities accurately and do not appear to have PH. In other words, plasma natriuretic peptides can be effectively used to exclude the presence of PH in clinical practice [6]. Both for patients with HFrEF and HFpEF, increasing PAP is associated with a poorer prognosis [20, 24].

## Monitoring pulmonary artery pressure: a new approach for heart failure management

The ESCAPE trial [30] investigated whether invasive haemodynamic monitoring—target pulmonary capillary wedge pressure (PCWP) of 15 mmHg and right atrial pressure of 8 mmHg—would improve outcome compared to clinical assessment alone for patients hospitalised with heart failure. The results were disappointing; haemodynamic monitoring did not increase days alive out of hospital or reduce plasma concentrations of B-type natriuretic peptides. A subsequent meta-analysis conducted to assess the impact of invasive haemodynamic assessment by pulmonary artery catheter on the management of critically ill patients (13 studies, > 5000 patients, including those enrolled in the ESCAPE trial), suggested no clinical benefit [31].

### The chronicle device [32]

The Chronicle device had a lead-mounted pressure sensor placed in the outflow tract of the RV. The lead was connected to a box, implanted subcutaneously in the pectoral area and containing the electronic components and power source. The pressure sensor used the principle of variable capacitance to provide measures of RV systolic and diastolic pressures and to assess the maximum rate of the pressure increase and decrease (max d*P*/d*t*) used to estimate the pulmonary artery diastolic (ePAPd) pressure that closely correlated with the pulmonary artery diastolic pressure (PAPd), measured invasively. A handheld radiofrequency wand, placed over the chest by the patient, interrogated the device and transmitted data remotely to healthcare professionals for review. Initially developed as a stand-alone device, the technology was subsequently incorporated into an implantable cardioverter defibrillator (ICD). The device measured pressures continuously but stored only a sample in memory that could be transmitted; this was usually the 8.5 min prior to each transmission. Patients could be instructed to rest or take exercise prior to transmitting data.

### Chronicle ICD system [33]

This device represented an evolution of the Chronicle, with the pressure-sensing system incorporated into an ICD. A single-chamber ICD lead was placed in the RV apex and an additional lead positioned in the RV outflow tract to measure pressures.

### CardioMEMS system [34]

CardioMEMS measures PAP using micro-electromechanical system (MEMS) technology and requires neither batteries nor leads. The sensor is 15-mm long and 3-mm wide and is permanently implanted in a distal branch of the PA via RHC. Two loops at the ends of the sensor serve as anchors and allow automatic sizing to the width of the vessel. The PA sensor consists of a three-dimensional coil and pressure sensitive capacitor. The coil electromagnetically couples the pressure sensitive capacitor to the electronics system, allowing the remote measurement of the resonant frequency then it is converted to a pressure measurement. Collection of haemodynamic data requires the patient to lie on an external pillow-like device which injects radiofrequency energy into the sensor, receives back signals to generate the waveform and transmits the data to a remote service facility that then relays the results to the patient’s healthcare provider**.** Only a few minutes of PA pressures are transmitted. Thus, although the sensor is always exposed to PA pressures, it is not currently possible to obtain 24 h pressures.

Compared to the Chronicle device, CardioMEMS does not require a surgical implantation procedure with the entailed risks and complications; CardioMEMS also measures PA pressure directly. However, a potential limitation of CardioMEMS is the difficulty in capturing data during exercise. Assessing PA pressure during exercise might detect sub-optimal control of congestion at an earlier stage.

## Systematic review and meta-analysis

In symptomatic patients with heart failure, monitoring of PAP using a wireless haemodynamic monitor system has been assigned a class IIb recommendation (level of evidence B) by the 2021 European Society of Cardiology (ESC) heart failure guidelines [35] in order to improve clinical outcomes. The current guidelines on heart failure, provided by the AHA/ACC/HFSA [36], were recently updated. Consistent with ESC guidelines, they assigned a class IIb recommendation for the use of PAP haemodynamic monitors, but they restricted this indication only to the patients in New York Heart Association (NYHA) functional class III who had previously been hospitalised for heart failure or had elevated plasma concentrations of natriuretic peptides. They also highlighted that PAP monitoring was of uncertain benefit in reducing the risk of subsequent heart failure hospitalisation. Earlier in 2022, the US Food and Drug Administration (FDA) extended the indications for the CardioMEMS PA pressure monitor to include patients with heart failure ‘who have either been hospitalized for heart failure in the previous year and/or have elevated natriuretic peptides’, opening the way to more widespread use of this technology.

To further evaluate the validity of these recommendations, we conducted a meta-analysis (graphical abstract) after the publication of the largest trial to date, GUIDE-HF trial [37].

Our primary and secondary outcomes were total heart failure hospitalisations and all-cause mortality, respectively. Full methods are shown in the supplementary material. Briefly**,** on the 22nd of September 2021**,** we searched Medline (PubMed), Embase (Ovid) and the Cochrane Central Register of Controlled Trials (CENTRAL) databases for randomised trials that investigated the use of implantable haemodynamic systems to monitor PAP in patients with heart failure.

**Fig. 1:**
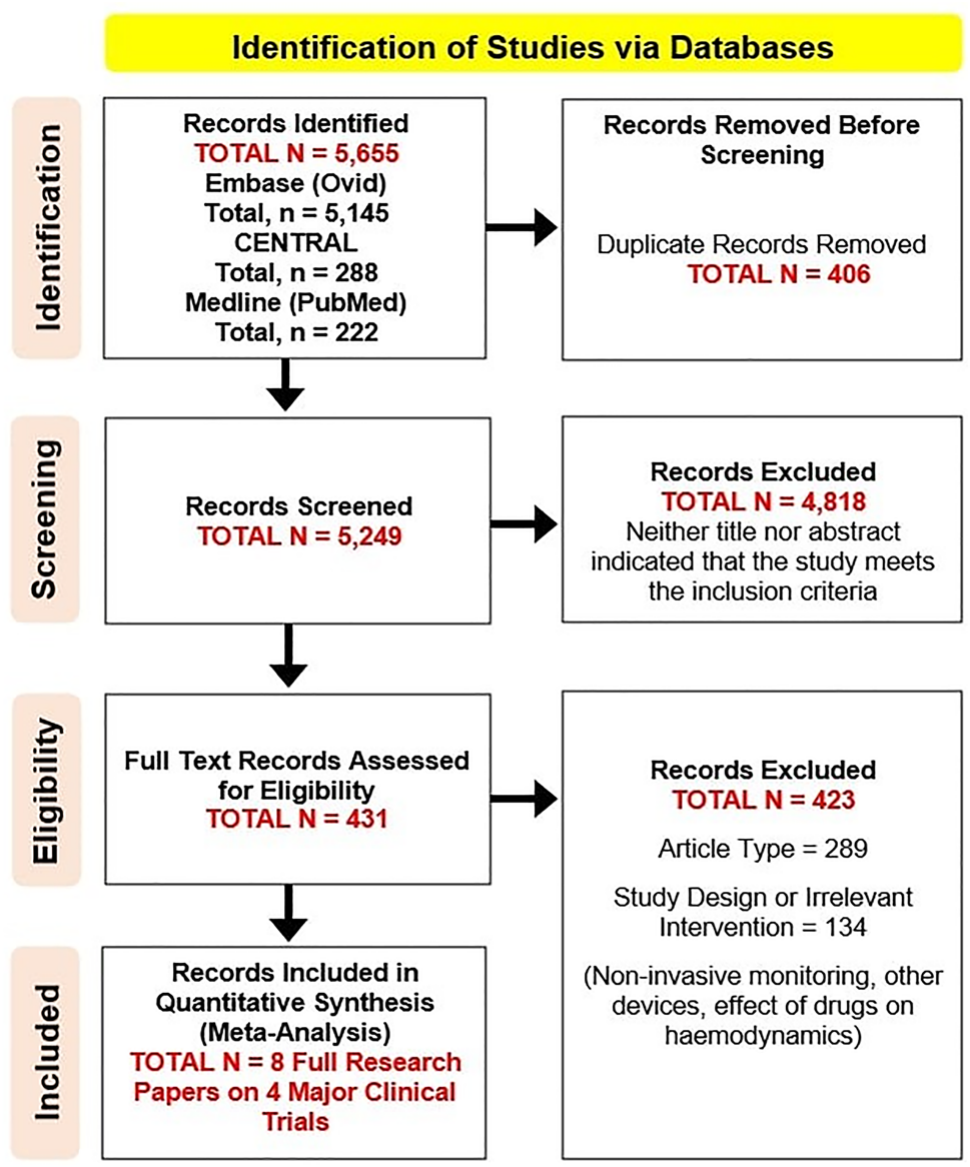
The identification process of the studies. PRISMA flow diagram of the studies retrieval and selection process used

**Fig. 2:**
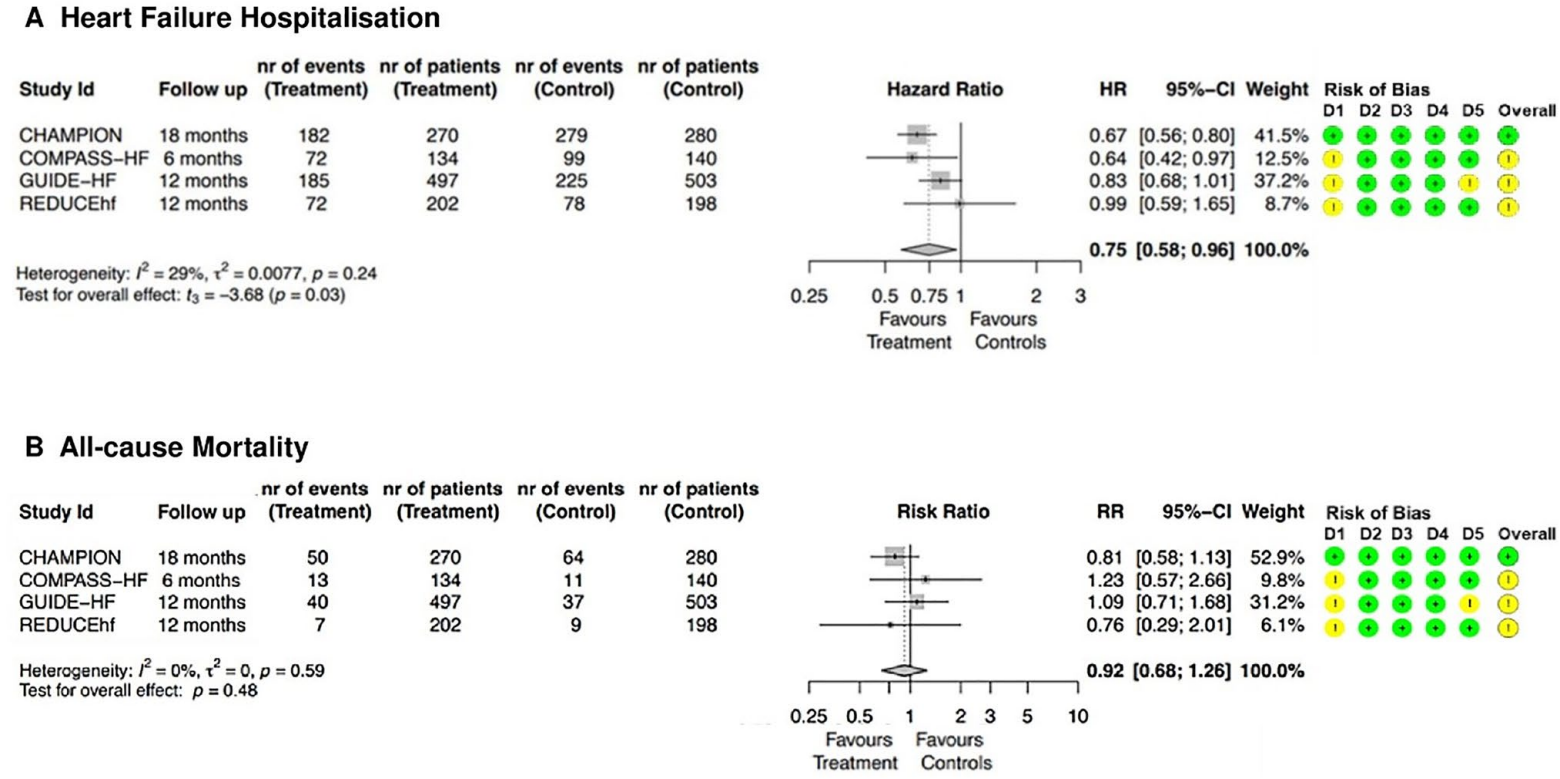
Primary outcomes: heart failure hospitalization (**A**) and all-cause mortality (**B**) at the longest follow-up available. Risk of bias was assessed for five domains: (D1) randomisation process, (D2) deviations from the intended interventions, (D3) missing outcome data, (D4) measurement of the outcome, (D5) selection of the reported result (green indicates low risk of bias and yellow indicates some concerns of bias)

Only fully peer-reviewed manuscripts written in English were considered for inclusion. After removing duplicates (*n* = 406), a further 4818 records were excluded by screening titles and abstracts; the remaining 431 articles were fully evaluated. We finally identified eight papers from four clinical trials: the CardioMEMS Heart Sensor Allows Monitoring of Pressure to Improve Outcomes in NYHA Class III Heart Failure Patients (CHAMPION, four papers) [38–41], the Chronicle Offers Management to Patients with Advanced Signs and Symptoms of Heart Failure (COMPASS-HF, two papers) [42, 43], the Reducing Decompensation Events Utilizing Intracardiac Pressures in Patients With Chronic Heart Failure (REDUCEhf, one paper) [44] and the haemodynamic-GUIDEd management of Heart Failure (GUIDE-HF, one paper) [37]. Figure 1 shows the results of our search strategy (PRISMA flow diagram), whilst table S1 (supplementary material) summarises the baseline characteristics of enrolled populations. All the trials we found were conducted in North America between 2008 and 2021 and all patients enrolled had a monitoring device implanted, regardless of allocation; however, the information acquired by the sensors were disclosed to physicians only for patients in the intervention arm. A key strategy that differentiated the design of CHAMPION and GUIDE-HF was the introduction of specific algorithms that guided the clinicians on the implementation of therapy according to haemodynamic readings. More detailed information about these trials are summarised in Table 2. Overall, compared to standard care, the use of implanted haemodynamic sensors reduced the risk of total heart failure hospitalisation by 25% (2224 patients; HR 0.75; 95% CI 0.58–0.96; *p* = 0.03) (Fig. 2A), using data from the longest follow-up available, but was not associated with a reduction in the risk of all-cause mortality (RR 0.92; 95% CI 0.68–1.26; *p* = 0.48) (Fig. 2B).

**Table 2:**
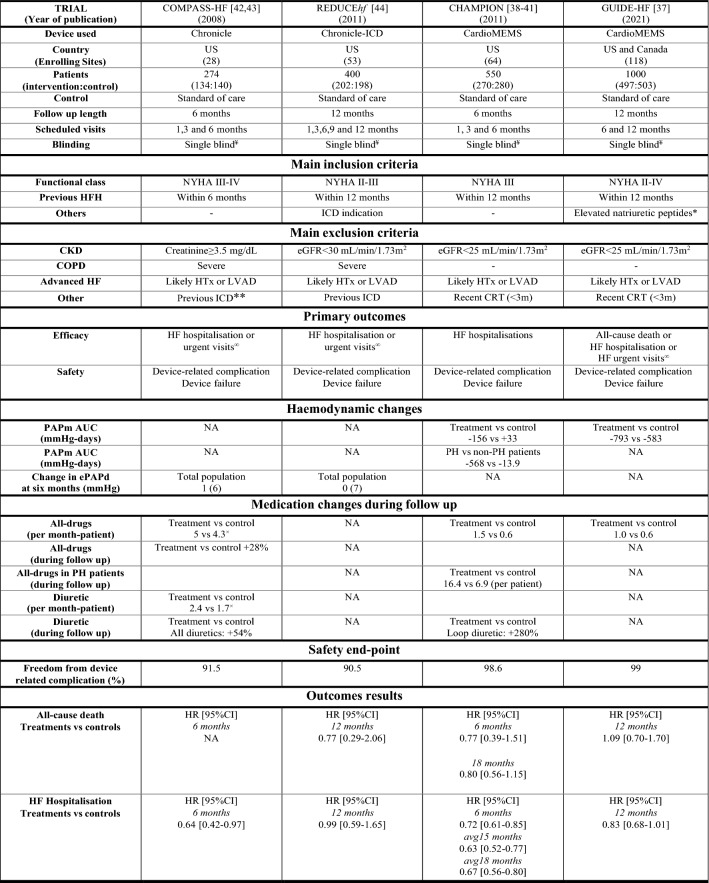
Key characteristics of the trials included in the meta-analysis

*AUC* area under the curve, *BMI* body mass index, *CKD* chronic kidney disease, *COPD* chronic obstructive pulmonary disease, *CRT* cardiac resynchronization therapy, *eGFR* estimated glomerular filtration rate, *HF* heart failure, *HFH* heart failure hospitalisation, *HR* hazard ratio, *HTx* heart transplantation, *ICD* implantable cardiac defibrillator, *LVAD* left ventricular assist device, *NYHA* New York Heart Association, *ePAPd* estimated diastolic pulmonary artery pressure, *PAPm* pulmonary artery mean pressure, *US* United States

^a^Only participants but not the investigators were blinded

^b^Patients without previous heart failure hospitalisation meat inclusion criteria if they report elevated natriuretic peptides in the 30 days prior to the consent (prespecified thresholds defined brain-type natriuretic peptide ≥ 250 pg/ml or amino-terminal pro-brain natriuretic peptide values ≥ 1000 pg/ml)

^c^If not compatible with the monitoring device

^d^Emergency room access evaluations following by intravenous diuretic therapy

^e^These data refer only to the HFpEF cohort

## Discussion

Considering the cost and complexity of haemodynamically guided monitoring for patients with heart failure, the results of randomised trial conducted, so far, are rather disappointing. Although haemodynamic monitoring might reduce hospitalisations for heart failure, the confidence intervals around the estimate are wide and the statistical certainty low. There are many potential explanations for why a reduction in hospitalisations and PA pressure might not translate into a reduction in mortality. Longer follow-up might be required for such small reductions in PA pressure to translate into reductions in mortality; more intense management to normalise PA pressure might lead to a greater reductions in hospitalisations and mortality but with the risk of more side effects. Perhaps the therapeutic algorithms are too cautious; perhaps current treatments are just not sufficiently effective [45, 46].

In order to improve management of heart failure by haemodynamic monitoring, measurements should be accurate in order to avoid false alerts and detect true ones and must be followed by changes in management to correct the perceived problem (a low or a high PA pressure). The optimal target PAP may differ from one patient to the next. A too low pressure due to intensification of heart failure therapy may lead to a fall in cardiac output, systemic arterial pressures and renal function. Patient engagement is essential. Unless patients are given the correct advice and follow management recommendations, haemodynamic monitoring will not be of any help. There is a long ‘delivery chain’ including the sensor, the patient taking the measurement, transmission to the service centre, relay on to the care team, the formulation of recommendations by the care team, the transmission of those recommendations back to the patient and the patient acting on the advice. There is a lot that could go wrong: the chain is only as strong as its weakest link.

In the COMPASS-HF [42] trial, heart failure therapies, particularly loop diuretics, were adjusted more frequently in patients assigned to the haemodynamic-guided arm than controls but trialists did not define an ePAPd target of treatment and, as a consequence, the mean ePAPd of the whole population remained high, at around 28 ± 7 mmHg throughout the entire study [42–44], suggesting either that investigators failed to recommend more intense treatment or that patients did not or could not (due to side effects) implement it. This failure is important, as a retrospective analysis using pooled data from three studies of the Chronicle programme (*n* = 790 patients) suggests that only a substantial reduction in ePAPd during follow-up reduced mortality; however, a decrease of 3 mmHg, or more, between baseline and 6 months was observed in less than 20% of patients [47–49].

In CHAMPION [38], haemodynamic-guided therapy was associated with a 28% reduction in heart failure hospitalisations, compared to the control group, during 6 months of follow-up, with similar encouraging results considering data from the entire follow-up (mean 18 months). CHAMPION provided instructions to investigators on how to modify treatment, mainly diuretics—followed by vasodilators, to achieve pre-specified ‘optimal’ haemodynamic readings. During follow-up, not only diuretics and vasodilators but also other neuro-hormonal antagonist therapies were adjusted more frequently in those assigned to active monitoring [39, 47]. Rather than responding to measurements thought to reflect an imminent problem, CHAMPION investigators attempted to adjust treatment constantly to maintain PAP as close to ideal as possible [50]. However, treatment guided by haemodynamic monitoring reduce PAPm by only ~ 5% in relative terms and only − 1.6 mmHg in absolute terms compared to no changes observed in the control group. Findings from CHAMPION also confirmed the close relationship between PH and adverse outcome [38]. Of the 537 patients enrolled and with complete baseline haemodynamic data, the 320 (59%) who met the criteria of Group II PH [8] had a higher rate of heart failure hospitalisations and greater risk of death than those without PH.

GUIDE-HF is the largest trial of haemodynamic monitoring conducted so far. By design, each patient was to be followed for only 12 months. Investigators were asked to contact participants twice a month for the first 3 months and then monthly until the end of the trial. Haemodynamic monitoring may provide less benefit when clinical care in the control group is frequent and of a high quality. Unfortunately, the COVID-19 pandemic struck when only about 40% of patients had completed 12 months of follow-up. An analysis [51] comparing findings prior and after the spread of COVID-19 pandemic was published recently, after our systematic search. The GUIDE-HF trial appeared to be on course for a positive result consistent with CHAMPION prior to COVID but then something remarkable happened. With the advent of COVID, PA pressures improved in the control group to the same extent as the intervention group. It appears that patients with heart failure during COVID might have become more adherent to their treatments and may have modified their lifestyle. Once the difference in pressures was lost, so was the difference in event rates. In a sense, this proves that better control of PA pressures is effective but questions whether we need frequent haemodynamic monitoring to achieve it. Perhaps monitoring every few months by a non-invasive approach, such as ultrasound, would be similarly effective. However, the reduction in PA pressures even prior to COVID was modest [45]. Both CHAMPION and GUIDE-HF show that we need more effective means of controlling PAP, either better implementation of existing interventions or new treatments.

The rate of hospitalisation in CHAMPION and GUIDE-HF appears to be much higher, both in the intervention group and in the control arm, compared to many other landmark trials of heart failure (Table S3, supplementary material). Differences in the characteristics of the population, in the design of the trial or the number of contacts between patients and physicians, during follow-up, both in the treatment and control arms, could explain the high rates of hospitalisation. Patients in GUIDE-HF may have been in a worse functional class than most other trials and had poorer renal function but they did not have a higher plasma NT-proBNP or annual mortality.

Although many patients with elevated PAP must have been enrolled in landmark trials of beta-blockers and angiotensin-converting enzyme inhibitors and angiotensin receptor blockers (ACE-I/ARBs), the effects of these therapies on PAP have been rarely evaluated. In animal models, mineralocorticoid receptor antagonists improve RV function and reverse PH, but studies in patients with heart failure and PH are lacking [52]. Indeed, no trial has ever evaluated the effects of loop diuretics on pulmonary pressures, even if they are the most commonly used drugs to decongest patients with evidence of elevated PAP.

Tran and colleagues [53] reported a rapid fall in mean (− 3.6 mmHg), systolic (− 6.5 mmHg) and diastolic (− 2.5 mmHg) PA pressures when treatment with ACE-I/ARBs was switched to sacubitril/valsartan in 18 patients with HFrEF with a CardioMEMS implant. Consistent with these results, Khan and colleagues [54] reported a reduction in PAPd (− 2.5 mmHg), PAPs (− 3.6 mmHg) and PAPm (− 3.2 mmHg) following initiation of treatment with sacubitril/valsartan in 13 patients with HFrEF who also had been implanted with a CardioMEMS device.

Sodium glucose cotransporter-2 inhibitors (SGLT2-Is) have recently been shown to be highly effective for the treatment of heart failure [55]. In a single-centre observational study, dapagliflozin reduced CardioMEMS PAPm from 42 ± 9 to 38 ± 10 mmHg, after seven days from initiation of the treatment [56]. In the EMBRACE-HF trial [57], 65 patients with heart failure (mean age 66 years, 97% on loop diuretics) and a CardioMEMS system (mean PAPd 22 mmHg) were randomised to empagliflozin or placebo. Compared to those assigned to placebo, empagliflozin 10 mg/day reduced the PAPd (averaged between 8 and 12 weeks, primary endpoint) by ~ 1.5 mmHg, regardless of heart failure phenotype. A greater proportion of patients assigned to empagliflozin also achieved a ≥ 20% reduction in plasma amino-terminal pro-brain natriuretic peptide values at 12 weeks (34% versus 7%; *p* = 0.01).

The real-world clinical experience with CardioMEMS provides the opportunity to evaluate the combined effects of intensification of treatment on PAP in patients with heart failure but also the long-term safety of this approach. Using data from a post-approval registry of 1200 patients with severe heart failure symptoms implanted with CardioMEMS, Shavelle and colleagues [58] showed that intensification of medications reduced PAPm during 1 year of observation in those with baseline PAPm ≥ 35 mmHg (− 4.8 ± 6.2 mm Hg), but when PAPm was < 25 mmHg at baseline, pressures rose (+ 1.5 ± 5.8 mm Hg). Patients with a baseline PAPm of 25–34 mmHg had an intermediate response (− 1.3 ± 5.0 mmHg).

These findings replicate those previously reported by Heywood and colleagues [59], who used de-identified PAP data from the first 2000 patients with heart failure implanted with a CardioMEMS who had at least 6 months of follow-up. They found that patients with a PAPm ≥ 35 mmHg at implantation had a fall in pressure (from 44 ± 7 to 37 ± 10 mmHg), whilst for those with a baseline PAPm < 25 mmHg (from 21 ± 3 to 22 ± 7 mmHg) or 25–34 mmHg (from 30 ± 3 to 29 ± 7 mmHg) pressures were unchanged.

Of the 5,500 CardioMEMS implanted in the US during the first 3 years after FDA approval, the reported rate of adverse events was relatively low (155 events; 2.8%), including PA injury (28 reports, 6 of which culminated in death), 46 sensor malfunction or migration (1%), 15 (0.3%) bleeding or infection at the vascular access and 5 pulmonary embolism or device thrombosis [60]. Experience from Germany [61] and the UK [62] confirms that tailoring treatment according to PAP using CardioMEMS is safe and feasible also in the European health care systems.

Uncertainty remains about the cost-effectiveness of the CardioMEMS. Further research is required to estimate, more accurately, the financial sustainability at scale [63]. Whether these devices should be restricted to patients with PH is uncertain. The devices are expensive and therefore may not be cost effective in sick patients with a short life expectancy nor in well-controlled patients who may have few events. There will be a ‘sweet spot’ where patients are neither too well nor too sick to benefit [64].

Technological developments now allow pulmonary artery pressure to be combined with other vital signs, to allow clinicians to individualise treatments with greater precision and without the need of clinical visits [65].

Most of the circulating blood volume is contained in the highly compliant venous system, which might buffer the impact of an increased circulating volume on PAPm. The CardioMEMS device can measure PCWP, which reflect left atrial pressure, but it appears the snapshots that the device takes may be less reliable and therefore treatment recommendations are not based on them. Devices implanted in the atrial septum to measure left atrial pressure are being investigated [66]. Monitoring venous capacity by ultrasound or other means might be an even better approach to detect and correct haemodynamic problems (both under- and over-filling) than measuring PAP [67, 68].

## Limitations

We were only able to access to published information, and not to individual patient data, which precluded more detailed analysis. Additional limitations include the heterogeneity of the devices used in the trials and changes in practice and patient demographics over time (the first patients were enrolled in COMPASS-HF in 2003, in CHAMPION in 2007 and in GUIDE-HF in 2018); patients in GUIDE-HF were, on average, a decade older than in CHAMPION (see supplementary Table S1).

## Conclusions

Monitoring pulmonary artery pressure in patients with heart failure reduces the risk of total heart failure hospitalisations, but not mortality. The results of our meta-analysis not only support recently updated professional guidelines but also highlight the need for further research before recommending widespread use of these currently costly technologies. Better patient selection, better patient engagement and education, better therapeutic algorithms, more ambitious haemodynamic targets and more effective and well-tolerated interventions to achieve them could yet make haemodynamic monitoring a cornerstone of care for patients with heart failure.

Further research is required to implement these therapeutic strategies and to identify patients more likely to benefit, thereby justifying the additional costs.

## Supplementary Information

Below is the link to the electronic supplementary material.

Supplementary file1 (DOCX 56 KB)
