# Supplementary material for: Implanted haemodynamic telemonitoring devices to guide management of heart failure: a review and meta-analysis of randomised trials
Source: Clin Res Cardiol. 2022 Oct 14;112(8):1007–19. doi: 10.1007/s00392-022-02104-0 (PMC9568893; doi:10.1007/s00392-022-02104-0)
Supplement: Supplementary file 1 — Supplementary file1 (DOCX 56 KB) [file 392_2022_2104_MOESM1_ESM.docx]

# Supplementary Online Content

**-Page 2:** methods.

**-Page 5:** search strategy.

**-Page 6:** *table S1.* Baseline characteristics of the trials populations.

**-Page 8:** *table S2.* Risk of bias assessment for outcomes of all-cause mortality and total heart failure hospitalisation.

**-Page 9:** *table S2.* Comparison between haemodynamic PAP monitoring trials to the landmark trials on heart failure.

**-Page 11:** references.

# Methods

*Search Strategy and Study Selection*

We searched Medline (PubMed), Embase (Ovid), the Cochrane Central Register of Controlled Trials (CENTRAL) databases for randomised trials on implantable haemodynamic devices to monitor PAP in patients with heart failure. The search strategy consisted of the definition of condition, the intervention applied and the study design including a combination of Medical Subject Headings (MeSH) and keywords restricted to titles and abstracts.

EC, AR and AI drafted a search strategy informed by a senior co-author (PP). The search was conducted on the 22^nd^ of September 2021 and screening of abstract and manuscripts was completed independently by EC, AR and AI. Disagreements were resolved by consensus, with the help of a fourth reviewer when required (PP). We included peer-reviewed or pre-print randomised trials that enrolled ambulatory patients with heart failure and compared haemodynamic-guided therapy, provided by an invasive PAP monitoring system vs standard of care. Trials assessing non-invasive telemonitoring, evaluating devices other than those used to monitor PAP or those evaluating the haemodynamic effects of medications in a treatment group were excluded. We did not include observational studies, reviews, meta-analysis, editorial or opinion articles and conference abstracts, as well as articles not written in English.

*Primary and secondary outcomes*

The primary outcome was total heart failure hospitalisation. Secondary outcome was all-cause mortality.

*Data Extraction and Risk*

Three review authors (EC, AR, AI) independently extracted data from included trials using an ad-hoc developed data collection form. Where available, we extracted the following data: first author, site of study, follow-up duration, main inclusion and exclusion criteria, patient characteristics (age and gender, comorbidities, symptoms and vital signs, blood results, treatments, hemodynamic data), and primary and secondary outcomes.

***Statistical Analysis***

For both the outcomes, the inverse variance random effect was used to obtain pooled estimates of intervention effect (Risk Ratio – RR for All-Cause mortality and Hazard Ratio – HR for total heart failure hospitalisation) with the associated 95% confidence interval (CI). The Restricted maximum-likelihood estimator was used to estimate the between study variance (tau squared). HR for REDUCEhf was extracted from Adamson et colleagues [1]. Effect estimates were graphically presented using Forest plots. Due to the small number of trials included, the Hartung and Knapp (HK) adjustment was used. We employed the I² statistic to measure the extent of unexplained heterogeneity: I² greater than 50% was considered a high degree of between-study heterogeneity. Meta-analysis was performed using the meta package in the statistical software R Version.

*Description of Included Trials and Baseline Characteristics*

We included data from four randomised trials that enrolled 2,224 patients in total. All trials were single-blinded and multi-centre; three trials were conducted in the USA [38,42,44] while GUIDE-HF [37] was also conducted in Canada. *Table 2* summarises the main characteristics of included trials and their major findings**.** Median age of trial participants ranged from 55 to 71 years, 66 % were male and prescription of loop diuretics was almost ubiquitous (95%). The baseline characteristics of the populations included in the four trials are shown in the *table S1* (supplementary material)*.*

*Risk of Bias Assessment*

Two authors (EC, AR) independently assessed risk of bias for primary and secondary outcomes using the Cochrane Risk of Bias 2 (RoB2) tool and the certainty of evidence for each outcome using the

GRADE approach. For the outcomes evaluated, CHAMPION [38] was judged to have low risk of bias in all domains, while REDUCEhf [44], COMPASS-HF [43] and GUIDE-HF [37] resulted in an overall judgment of some concerns (*table S2*, supplementary material). Despite the single blind design of all included trials, deviations from intended interventions due to potential patient unblinding or favouring more healthcare-patient interactions for the intervention group were unlikely due to rigorous measures implied to maintain patient blinding and ensure balanced frequency of healthcare-patient contact within both groups. GRADE assessment for mortality pooled estimate reveals moderate certainty due to likely imprecision attributed to low total number of events (231), while total heart failure hospitalisation benefit associated with use of implantable heamodynamic monitoring devices is assessed at high certainty level.

# Search strategy

Condition:

Heart failure OR Congestive heart failure OR Chronic heart failure OR Congestion OR Hemodynamic congestion

Intervention:

Pulmonary artery remote monitoring OR Pulmonary artery continuous monitoring OR Implantable pulmonary artery pressure monitor OR Pulmonary artery diastolic pressure OR Pulmonary artery pressure monitoring OR pulmonary artery pressure-guided management OR pulmonary arterial devices OR pulmonary arterial device OR Right heart pressure guided OR Right heart pressure monitoring OR Heart failure continuous monitoring OR Heart failure remote monitoring OR Heart continuous remote monitoring OR hemodynamic monitoring device OR haemodynamic monitoring device OR hemodynamic monitoring devices OR haemodynamic monitoring devices OR Haemodynamic monitoring system OR Haemodynamic monitoring systems OR Hemodynamic monitoring system OR Hemodynamic monitoring systems OR Wireless implantable sensor OR Wireless implantable sensors OR Remote monitoring OR Remote sensing technology OR Home telemonitoring OR Remote telemonitoring OR invasive monitoring devices OR invasive monitoring device OR invasive remote monitoring OR CardioMEMS OR Cordella

Study Design:

Randomised Controlled Trial OR Randomized Controlled Trial OR Randomised Controlled Trials OR Randomized Controlled Trials OR Randomised Clinical Trial OR Randomized Clinical Trial OR Randomised Clinical Trials OR Randomized Clinical Trials OR Controlled Trial OR Controlled Study OR Trial OR Randomisation OR Randomization OR Controlled Clinical Trial OR Clinical Trial.

| **Table S1: baseline characteristics of the trials populations** | | | | | |
| --- | --- | --- | --- | --- | --- |
| **Variable^∞^** | **Group** | **CHAMPION**  **[38-41]**  **(N=550)** | **REDUCE*hf***  **[44]**  **(N=400)** | **COMPASS-HF**  **[42]**  **(N=274)** | **GUIDE-HF**  **[37]**  **(N=1000)** |
| **Demographic, history and laboratory assessment** | | | | | |
| **Age** | Intervention Control | 61 (13)  62 (13) | 55 (15)  55 (15) | 58 (14)  58 (13) | 71 (64 – 76)  70 (64 – 77) |
| **Men (%)** |  | 73 | 69 | 65 | 62 |
| **Hypertension (%)** |  | 78 |  |  |  |
| **DM (%)** |  | 49 |  |  | 50 |
| **CAD (%)** |  | 70 | 45 | 46 | 40 |
| **AF (%)** |  | 46 |  | 39 | 59 |
| **NYHA II (%)** |  | 0 | 49 | 0 | 30 |
| **NYHA III (%)** |  | 100 | 51 | 85 | 65 |
| **NYHA IV (%)** |  | 0 | 0 | 15 | 5 |
| **BMI** | Intervention | 31 (7) | Total population: | Total population: | 32 (27 – 38) |
| kg/m^2^ | Control | 31 (7) | 31 (7) | 36 (9) | 33 (28– 39) |
| **Creatinine** | Intervention | 124 (44) |  |  | 121 (97-150) |
| µmol/L | Control | 124 (44) |  |  | 124 (97-159) |
| **eGFR** | Intervention | 60 (23) | Total population: | Total population: | 51 (39.3-65.3) |
| ml/min/1.73m^2^ | Control | 62 (23) | 72 (24) | 56 (24) | 49 (38.1-64.8) |
| **BNP** | Intervention |  |  |  | 267 (133-612) |
| pg/mL | Control |  |  |  | 292 (139-586) |
| **NTproBNP** | Intervention |  |  |  | 1480 (686-2743) |
| pg/mL | Control |  |  |  | 1274 (661-2318) |
| **Cardiac haemodynamics** | | | | | |
| **Heart Rate** | Intervention | 72 (±13) | Total population: | Total population: | 72 (65-82) |
| beats/min | Control | 73 (±12) | 76 (14) | 77 (14) | 72 (65-82) |
| **SBP** | Intervention | 121 (±23) | Total population: | Total population: | 120 (108-132) |
| mmHg | Control | 123 (±21) | 116 (21) | 112 (19) | 120 (108-132) |
| **DBP** | Intervention | 72 (±13) | Total population: | Total population: | 68 (60-76) |
| mmHg | Control | 72 (±13) | 72 (13) | 68 (11) | 68 (61-76) |
| **PAPs** | Intervention | 44 (±14) |  |  | 44 (35-52) |
| mmHg | Control | 45 (±15) |  |  | 42 (35-53) |
| **PAPd** | Intervention | 19 (±8) |  |  | 18 (14-23) |
| mmHg | Control | 19 (±8) |  |  | 18 (13-24) |
| **PAPm** | Intervention | 29 (±10) |  |  | 28 (22-35) |
| mmHg | Control | 30 (±10) |  |  | 29 (22-35) |
| **PCWP** | Intervention | 16 (±6) |  |  | 16 (11-23) |
| mmHg | Control | 15 (±7) |  |  | 17 (12-22) |
| **Cardiac Index** | Intervention | 2.1 (±0.6) |  |  | 2.1 (1.8-2.6) |
| L/min/m^2^ | Control | 2.2 (±0.6) |  |  | 2.1 (1.7-2.5) |
| **PVR** | Intervention | 2.9 (±2.0) |  |  |  |
| Wood units | Control | 2.7 (±1.8) |  |  |  |
| **ePAPd** | Intervention |  | 22.4 (±8) | Total population |  |
| mmHg | Control |  | 22.3 (±7.2) | 27 (8) |  |
| **Treatment** | | | | | |
| **BBs (%)** |  | 91 | 96 | 82 | 89 |
| **ACE-I/ARBs/ARNi (%)** |  | 78 | 92 | 83 | 93 |
| **MRAs (%)** |  | 42 |  |  | 45 |
| **Loop Diuretics (%)** |  | 92 | 95* | 96* | 95 |
| **Hydralazine (%)** |  | 13 |  |  | 16 |
| **Nitrates (%)** |  | 22 |  |  | 20 |
| **ICD only (%)** |  | 34 | Excluded |  | 42 |
| **CRT (%)** |  | 35 | Excluded |  | 29 |

**Abbreviations:** ACE-I: angiotensin-converting enzyme inhibitor; AF: atrial fibrillation; ARBs: angiotensin II receptor blockers; ARNi: angiotensin receptor neprilysin inhibitor; BBs: beta blockers; BMI: body mass index; BNP: brain- type natriuretic peptide; CAD: coronary artery disease; CRT: cardiac resynchronization therapy; DM: diabetes mellitus; DPB: diastolic blood pressure; eGFR: estimated glomerular filtration rate; ICD: implanted cardioverter defibrillator; MRAs: mineralocorticoid receptor antagonists; NT-proBNP: amino terminal pro-brain natriuretic peptide; NYHA: New York heart association; PAPd: pulmonary artery diastolic pressure; ePAPd: estimated pulmonary artery diastolic pressure; PAPm: pulmonary artery mean pressure; PAPs: pulmonary artery systolic pressure; PCWP: pulmonary capillary wedge pressure; PVR: pulmonary vascular resistance; SBP: systolic blood pressure. Data are n (%), mean ± standard deviation or median (interquartile range).

| **Trial** | **Randomisation Process** | **Deviations from the Intended Interventions** | **Missing Outcome Data** | **Measurement of the Outcome** | **Selection of the Reported Result** | **Overall** |
| --- | --- | --- | --- | --- | --- | --- |
| **CHAMPION 2011** | Low | Low | Low | Low | Low | Low |
| **REDUCE*hf* 2011** | Some Concerns | Low | Low | Low | Low | Some Concerns |
|  | No information on randomization method and allocation concealment . | | | | | |
| **COMPASS-HF 2008** | Some Concerns | Low | Low | Low | Some Concerns | Some Concerns |
|  | No information on randomization method and allocation concealment. No published pre-specified statistical analysis plan/protocol. | | | | | |
| **GUIDE-HF 2021** | Some concerns | Low | Low | Low | Low | Some concerns |
|  | No information on allocation concealment | | | | | |

**Table S2.** Risk of bias assessment for outcomes of total heart failure hospitalisation and all-cause mortality.

| **Table S3. Comparison between haemodynamic PAP monitoring trials to the landmark trials on heart failure** | | | | | | | | |
| --- | --- | --- | --- | --- | --- | --- | --- | --- |
|  | **PARADIGM-HF**  **[2]** | **DAPA-HF**  **[3]** | **EMPEROR**  **[4]** | **VICTORIA**  **[5]** | **GALACTIC**  **[6]** | **GALACTIC**  **[7]**  **(post-hoc analysis of severe HF)** | **CHAMPION**  **[38]**  **(prolonged FU)*** | **GUIDE-HF**  **[37]** |
| Intervention tested | Sacubitril/Valsartan | Dapagliflozin | Empagliflozin | Vericiguat | Omecamtiv | Omecamtiv | PAP-guided | PAP-guided |
| Number of patients | 8401 | 4744 | 3730 | 5050 | 8232 | 2258 | 550 | 1000 |
| Median follow-up, month | 27 | 18 | 16 | 11 | 22 | 22 | 18 | 11 |
| Year of publication | 2014 | 2019 | 2020 | 2020 | 2021 | 2021 | 2011 | 2021 |
| **Trial characteristics** | | | | | | | | |
| Comparator | Enalapril | Placebo | Placebo | Placebo | Placebo | Placebo | No PAP-guided | No PAP-guided |
| Age (years) | 64 | 62 | 67 | 67 | 64 | 64 | 62 | 70 |
| Male sex (%) | 88 | 87 | 76 | 76 | 89 | 79 | 73 | 63 |
| LVEF (%) | 29 | 31 | 27 | 29 | 26 | 23 | 21 | 39 |
| eGFR ml/min/1.73 m^2^ | 68 | 66 | 62 | 61 | 59 | 55 | 61 | 50 |
| AF (%) | 37 | 40 | 37 | 45 | 27 | 32 | 46 | 59 |
| NT-proBNP pg/ml | 1612  (885-3229) | 1437  (857-2648) | 1907  (1115-3476) | 2816  (N/A) | 2001  (990-4083) | 2796  (1448-5785) | N/A | 1277  (667-2530) |
| NYHA III-IV (%) | 25 | 33 | 25 | 41 | 47 | 100 | 100 | 70 |
| **HR (95% CI) for key outcomes** | | | | | | | | |
| All-cause mortality | 0.84  (0.76-0.93) | N/A | 0.92  (0.77-1.10) | 0.95  (0.84-1.07) | 1.00  (0.92-1.09) | 1.00  (0.92-1.06) | 0.80  (0.55-1.15) | 1.09  (0.70-1.7) |
| Cardiovascular death | 0.80  (0.71-0.89) | 0.82  (0.69-0.98) | 0.92  (0.75-1.12) | 0.93  (0.81-1.06) | 1.01  (0.92-1.11) | 0.88  (0.75-1.03) | N/A | N/A |
| First HF  hospitalisation | 0.79  (0.71-0.89) | N/A | N/A | 0.90  (0.81-1.00) | 0.95  (0.87-1.03) | 0.84  (0.74-0.97) | N/A | N/A |
| Recurrent HF hospitalisation | N/A | 0.70  (0.59-0-83) | 0.69  (0.59-0.81) | 0.91  (0.84-0.99) | N/A | N/A | 0.67  (0.55-0.80) | 0.83  (0.68-1.01) |
| **Annualized event rate, n events per 100 patient at risk (control arm versus treatment group)** | | | | | | | | |

| HF hospitalisation or  cardiovascular death | N/A | 15 vs 11 | 21 vs 16 | 38 vs 34 | N/A | 43 vs 34 | 82 vs 57 | N/A |
| --- | --- | --- | --- | --- | --- | --- | --- | --- |
| Absolute rate reduction | N/A | 4 | 5 | 4 | N/A | 9 | 25 | N/A |
| Cardiovascular death | 7 vs 6 | 8 vs 6 | 8 vs 8 | 14 vs 13 | 11 vs 11 | 17 vs 15 | N/A | N/A |
| Absolute rate reduction | 1 | 2 | 0 | 1 | 0 | 2 | N/A | N/A |
| First HF  hospitalisation | 7 vs 6 | N/A | N/A | 30 vs 26 | 19 vs 18 | 31 vs 26 | N/A | N/A |
| Absolute rate reduction | 1 | N/A | N/A | 4 | 1 | 5 | N/A | N/A |
| Recurrent HF  hospitalisation | N/A | 14 vs 10 | 16 vs 11 | 42 vs 38 | N/A | N/A | 66 vs 45 | 50 vs 41 |
| Absolute rate reduction | N/A | 4 | 5 | 4 | N/A | N/A | 21 | 9 |

Values are given as percentage, mean, median and interquartile range. **Abbreviations:** AF: atrial fibrillation; eGFR: estimated glomerular filtration rate; HF: heart failure; LVEF: left ventricular ejection fraction; N/A: not available; NT-proBNP: N terminal pro brain natriuretic peptide; NYHA: New York heart association. PAP: pulmonary artery pressure. *These data are referring to the sustained follow-up of 18 months from CHAMPION trial.

# References

1. Adamson PB, Ginn G, Anker SD, Bourge RC, Abraham WT. Remote haemodynamic-guided care for patients with chronic heart failure: a meta-analysis of completed trials. Eur J Heart Fail 2017;**19**:426-433. DOI: 10.1002/ejhf.638.
2. McMurray JJ, Packer M, Desai AS, Gong J, Lefkowitz MP, Rizkala AR at al. Angiotensin- neprilysin inhibition versus enalapril in heart failure. N Engl J Med. 2014 Sep 11;371(11):993-1004. DOI: 10.1056/NEJMoa1409077. Epub 2014 Aug 30.
3. McMurray JJV, Solomon SD, Inzucchi SE, Køber L, Kosiborod MN, Martinez FA et al. Dapagliflozin in Patients with Heart Failure and Reduced Ejection Fraction. N Engl J Med. 2019 Nov 21;381(21):1995-2008. doi: 10.1056/NEJMoa1911303. Epub 2019 Sep 19.
4. Packer M, Anker SD, Butler J, Filippatos G, Pocock SJ, Carson P at al. Cardiovascular and Renal Outcomes with Empagliflozin in Heart Failure. N Engl J Med. 2020 Oct 8;383(15):1413-1424. doi: 10.1056/NEJMoa2022190. Epub 2020 Aug 28.
5. Armstrong PW, Pieske B, Anstrom KJ, Ezekowitz J, Hernandez AF, Butler J et al. Vericiguat in Patients with Heart Failure and Reduced Ejection Fraction. N Engl J Med. 2020 May 14;382(20):1883-1893. doi: 10.1056/NEJMoa1915928. Epub 2020 Mar 28.
6. Teerlink JR, Diaz R, Felker GM, McMurray JJV, Metra M, Solomon SD et al. Cardiac Myosin Activation with Omecamtiv Mecarbil in Systolic Heart Failure. N Engl J Med. 2021 Jan. 4;384(2):105-116. DOI: 10.1056/NEJMoa2025797. Epub 2020 Nov 13.

7. Felker GM, Solomon SD, Claggett B, Diaz R, McMurray JJV, Metra M at al. Assessment of Omecamtiv Mecarbil for the Treatment of Patients With Severe Heart Failure: A Post Hoc Analysis of Data From the GALACTIC-HF Randomized Clinical Trial. JAMA Cardiol. 2022 Jan 1;7(1):26- 34. DOI: 10.1001/jamacardio.2021.4027. PMID: 34643642; PMCID: PMC8515258.
